# Low Emissions and Delay Optimization for an Isolated Signalized Intersection Based on Vehicular Trajectories

**DOI:** 10.1371/journal.pone.0146018

**Published:** 2015-12-31

**Authors:** Ciyun Lin, Bowen Gong, Xin Qu

**Affiliations:** 1 State Key Laboratory of Automobile Dynamic Simulation, Jilin University, Changchun, 130022, China; 2 College of Transportation, Jilin University, Changchun, 130022, China; Beihang University, CHINA

## Abstract

A traditional traffic signal control system is established based on vehicular delay, queue length, saturation and other indicators. However, due to the increasing severity of urban environmental pollution issues and the development of a resource-saving and environmentally friendly social philosophy, the development of low-carbon and energy-efficient urban transport is required. This paper first defines vehicular trajectories and the calculation of vehicular emissions based on VSP. Next, a regression analysis method is used to quantify the relationship between vehicular emissions and delay, and a traffic signal control model is established to reduce emissions and delay using the enumeration method combined with saturation constraints. Finally, one typical intersection of Changchun is selected to verify the model proposed in this paper; its performance efficiency is also compared using simulations in VISSIM. The results of this study show that the proposed model can significantly reduce vehicle delay and traffic emissions simultaneously.

## Introduction

Road-based traffic emissions produce a large amount of suspended particulate matter in the air of urban cities. Traffic emissions have been shown to be the primary source of fog and haze in cities; these pollutants can cause significant problems, including reduced traffic visibility and decreased immune response in the respiratory systems of humans, etc. The studies show that the traffic fuel consumption has become an importation part of petroleum consumption and its emissions have turned a major source of air pollution[1–3]. And vehicles idling at signalized intersections often result in an increase in fuel consumption and emissions of pollutants, including CO, HC+NO_x_, PM and other noxious gases. Thus researchers developed many models and traffic control methods to study the traffic emissions and to reduce vehicles idling at signalized intersection. The-state-of-the-art study for traffic emissions is to explore the influences of the vehicle’s fuel consumption and exhaust emissions on the trip under different traffic conditions[4–12]. For example, researches developed car-following models to explore the impacts of the vehicle’s fuel consumption and exhaust emissions[13–16]. Some researches focus on studying traffic phenomena to developed traffic flow models or new technologies to enhance traffic safety and reduce fuel consumption and traffic emissions [17–26]. Traffic signal timing and traffic states of the intersection impacts on fuel consumption and emission are studied and evaluated in the literature[27–32]. In the literature, the importance of traffic signal timing optimization is well established by several studies conducted as a result of the increase serious traffic problems. The studies developed control models or methods to explore the complex traffic phenomena and to optimize the signal timing parameters by considering temporal, spatial, or temporal-spatial costs to vehicles and travelers, such as vehicle delay, queue length, saturation, etc[33–42]. However, development of an optimization model considering both traffic performance and traffic emissions has not received the same level of attention compared with the studies of explore the relationship of fuel consumption and emission and the studies of optimal traffic signal timing to maximize the intersection’s traffic capacity while minimizing traffic delay[43–48]. Although the number of studies about optimal traffic signal timing based on emission and delay is limited for regular traffic signal control, there is an agreement on signal timing optimization play an important role in traffic flow management by reducing traffic delay and emission[49–51].

Intelligent transportation systems (ITS) have the positive effect on improving traffic operation efficiency, alleviating traffic congestion, ensuring traffic safety and reducing traffic emissions and so on. Therefore, the state and governments at all levels take serious attitude towards the development and planning strategy of ITS. The governments have put forward a series of policies to stimulate its development as to maintain the sustainability of urban traffic systems. Synchronously, China aims to build a resource-conserving and environmentally friendly society, one of the earlier and most significant works focusing on building low-carbon and energy-saving systems for transportation system. So that determining how to optimize traffic flow in an urban traffic network with low emissions and delay is the primary goal of both ITS and low-carbon and energy-saving systems.

Therefore, the goal of this study is to optimize the traffic signal control parameters to minimize both the emissions and the delay of vehicles. The parameter VSP (Vehicle Specific Power) is used to calculate the traffic emissions of a vehicle based on vehicular trajectory. In addition, the relationship between traffic emissions and delay is quantified using regression analysis. Next, an optimization model for a traffic signal control system is established, including the control variables, constraint variables (such as traffic emission and delay) and target variables.

## Methods

### Vehicular trajectory analysis

**Fig 1:**
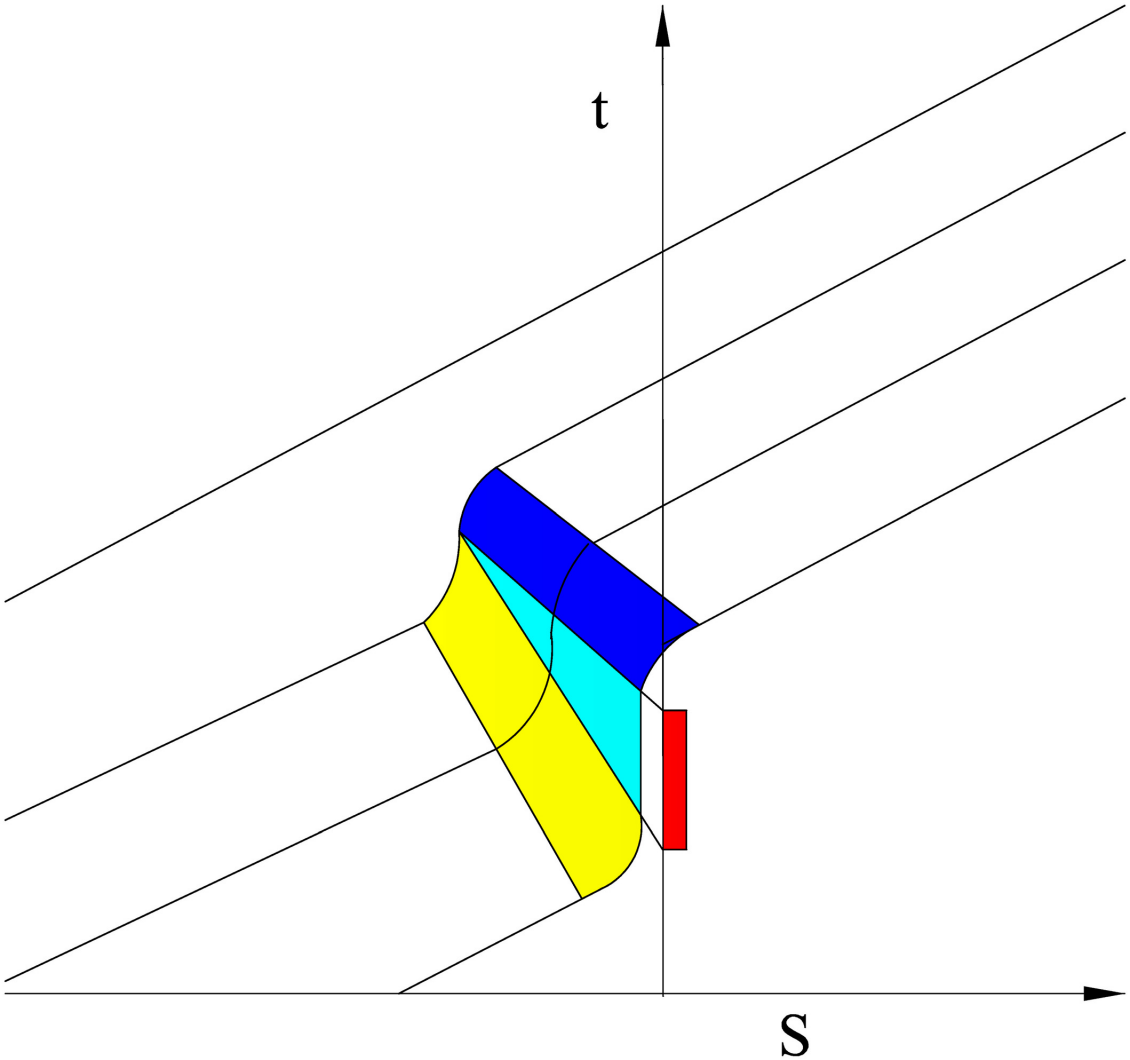
Vehicle trajectory diagram.

When a vehicle approaches a signalized intersection, the movement of the vehicle can be divided into four steps: constant speed, deceleration, queuing and acceleration. As shown in Fig 1, the red area represents the period during which the red signal is illuminated; the yellow area represents the period when the vehicle is decelerating; the light blue area represents the queuing period; and the dark blue area represents the period when the vehicle is accelerating.

The deceleration process can be described as follows:

$$S_{d}(t)=v_{f}t-0.5a_{d}t^{2}$$ (1)

$$t_{d}=\frac{v_{f}}{a_{d}}$$ (2)

$$S_{d}=S_{d}(t_{d})$$ (3)

where *S*_*d*_(*t*) is the distance function corresponding to the deceleration of the vehicle, *v*_*f*_ is the free-flow speed, *a*_*d*_ is the acceleration when the vehicle is decelerating and *t*_*d*_ is the duration of the vehicle’s deceleration.

The acceleration process can be described by Eq 4:

$$a_{a}(t)=\alpha \cdot v_{a}(t)+b$$ (4)

where $a_{a}(t)=\frac{\partial^{2}S_{a}(t)}{\partial t^{2}}$ is the acceleration of the accelerating vehicle, $v_{a}(t)=\frac{\partial S_{a}(t)}{\partial t}$ is the speed of the accelerating vehicle at time t, *S*_*a*_(*t*) is the distance function of the accelerating vehicle, *α* and *b* are the adjustment coefficients.

The initial conditions of the vehicle’s movement are given as follows. Setting *v*_*a*_(0) = 0 and *S*_*a*_(0) = 0, the following equations can be obtained:

$$a_{a}(t)=be^{at}$$ (5)

$$v_{a}(t)=\frac{b}{a}(e^{at}-1)$$ (6)

$$S_{a}(t)=\frac{b}{a^{2}}(e^{at}-at-1)$$ (7)

The duration and the distance of the accelerating vehicle can be calculated respectively as follows:

$$t_{a}=a_{a}(t)\cdot v_{a}^{-1}(t)$$ (8)

$$S_{a}=S_{a}(t_{a})$$ (9)

From the time and distance functions as the vehicle approaches the signalized intersection, the threshold of the vehicle delay *D*_*c*_ can be calculated using Eqs 1 through 9, resulting in Eq 10:

$$D_{c}=(t_{d}-\frac{S_{d}}{v_{f}})+(t_{a}-\frac{S_{a}}{v_{f}})$$ (10)

### Traffic emissions calculation

Many calculation models for vehicular emissions have been developed to date, both at home and abroad. For example, the MOBILE model and the EMFAC model are based on the driving cycle, which does not consider the engine load effect on the emissions[51–53]. The MEASURE model is based on the running state and thus does not consider the effect of the road level on the emissions[32]. The emissions model based on fuel consumption cannot describe the effects of the vehicle’s movement on its emissions[42]. Based on road tests, the MOVES model was built, and a more accurate microscopic simulation was developed. However, the MOVES model is not applicable in China[50]. Therefore, a more suitable emissions model is required for China's road traffic. VSP is defined as the output power when a vehicle’s engine moves one ton of mass, which best approximates the parameters of the real situation. VSP can effectively depict the real vehicular driving characteristics present on a road.

The derivation process of the VSP for vehicles can be represented by Eq 11 [54]:

$$VSP=\frac{\frac{d}{d_{t}}(KE+PE)+p_{f}+0.5\rho_{a}C_{D}A{\left(v+v_{w}\right)}^{2}v}{m}$$ (11)

where $\frac{d}{d_{t}}(KE+PE)$ is the power required to change the kinetic/potential energy; *p*_*f*_ *v* is the power required to overcome rolling resistance; 0.5*ρ*_*a*_ *C*_*D*_ *A*(*v*+*v*_*w*_)^2^ *v* is the power required to overcome aerodynamic drag; *KE* is the kinetic energy of the vehicle; *PE* is the potential energy of the vehicle; *p*_*f*_ is the rolling resistance; *m* is the mass of the vehicle (*kg*); *v* is the vehicle speed (*m*/*s*); *v*_*w*_ is the wind speed of the vehicle in the windward direction; *C*_*D*_ is the wind resistance coefficient, which is a dimensionless quantity; *A* is the cross-sectional area of the vehicle (*m* ^2^); and *ρ*_*a*_ is the air density.

For the kinetic and potential energies, the derivatives at time *t* can be obtained as follows:

$$VSP=v[a(1+\varepsilon_{i})+g(dg)+C_{R}g]+0.5\rho_{a}\frac{C_{D}A}{m}{\left(v+v_{w}\right)}^{2}v$$ (12)

The calculation of the VSP can be represented by Eq 13:

$$VSP=0.3227va+0.0954v+0.0000272v^{3}$$ (13)

VSP is divided into different sections based on a given interval. Next, the average of all instantaneous emissions rates is calculated in each region as the emissions rate of this interval; this can effectively mitigate the discrete nature of the data and forecast vehicular emissions accurately using parameters based on the VSP.

Ref. [54] describes the relationship between VSP and the pollutant emissions rate, as shown in Table 1. This study uses VSP interval division and the corresponding pollutant emissions rate to calculate the traffic emissions of signalized intersections.

**Table 1:** VSP interval division and pollutant emissions rate.

VSP	NO_x_ (g/s)	VOC (g/s)	CO (g/s)
-20	0.00031	0.0033	0.02111
-19	0.00165	0.00276	0.01949
-18	0.00013	0.00248	0.02597
-17	0.00092	0.00215	0.02169
-16	0	0.00141	0.02173
-15	0.00015	0.0017	0.01875
-14	0.00068	0.00261	0.01864
-13	0.00042	0.00167	0.017
-12	0.00027	0.00119	0.01602
-11	0.00048	0.00158	0.02227
-10	0.00045	0.00129	0.0226
-9	0.00019	0.00149	0.0246
-8	0.00027	0.00218	0.02187
-7	0.00035	0.00157	0.02212
-6	0.00019	0.00196	0.02437
-5	0.00039	0.00152	0.02378
-4	0.00046	0.00168	0.03078
-3	0.00026	0.0022	0.029
-2	0.00027	0.00237	0.02226
-1	0.00043	0.00173	0.0255
0	0.00011	0.00082	0.00582
1	0.00053	0.00292	0.04613
2	0.00184	0.00463	0.06289
3	0.00302	0.00458	0.06795
4	0.00402	0.0046	0.07323
5	0.00421	0.00548	0.0816
6	0.00496	0.0059	0.08184
7	0.00586	0.00785	0.09112
8	0.00736	0.00663	0.09895
9	0.0083	0.00829	0.00829
10	0.00923	0.00824	0.11238
11	0.01178	0.00936	0.11265
12	0.0125	0.00921	0.14685
13	0.01402	0.01045	0.13741
14	0.01491	0.00917	0.15765
15	0.01643	0.01207	0.16011
16	0.01878	0.0126	0.16208
17	0.01924	0.01456	0.15508
18	0.02129	0.01438	0.14174
19	0.02199	0.01641	0.18483
20	0.02203	0.01501	0.16303

The vehicle emissions model based on the VSP distribution shown above can be expressed by Eq 14:

$$MOE_{e,j}=\sum_{i=1}^{n}f\left(p_{i}\right)\cdot R\left(p_{i,j}\right)$$ (14)

where *MOE*_*e*,*j*_ is the *j* ^*th*^ type of traffic emission along a route; *i* is the travel time (*i* = 1,2,3…,*n*); *p*_*i*_ is the VSP value at time *i*; *f*(*p*_*i*_) is the VSP distribution at time *i* when the VSP value is *p*_*i*_; and *R*(*p*_*i*_,*j*) is the *j* ^*th*^ type of traffic emission rate when the VSP value is *p*_*i*_ at time *i*.

As a result, the vehicle emissions along the given route can be calculated based on the VSP distribution and the traffic emissions calculation model.

Different pollutants will produce different effects. This study uses the weighted average method to synthesize the different pollutants, and the influence of the unit is eliminated by normalized processing. Next, the expert scoring method is used to determine the weights of each indicator, whose values are 0.15, 0.7 and 0.15[55]. Thus, the total traffic emissions described by Eq 15:

$$E=0.15E_{VOC}+0.70E_{CO}+0.15E_{NO_{X}}$$ (15)

where *E*_*VOC*_ is the emission of volatile organic compounds (VOC); *E*_*CO*_ is the emission of carbon monoxide (CO); $E_{NO_{X}}$ is the emission of nitrogen oxides (NO_x_).

### Relationship between delay and emissions

Considering a real traffic situation at an intersection, this study sets the free flow speed of the vehicles before entering the intersection to be *v*_*f*_ = 70*km*/*h*. The acceleration and deceleration functions of the vehicles are based on the method proposed in VISSIM when the speed is lower than 70 mph [48]:

$$a_{d}\left(t\right)=2.74m/s^{2}$$ (16)

$$a_{a}\left(t\right)=-0.04023v_{a}\left(t\right)+3.5m/s^{2}$$ (17)

This study quantifies the relationship between the control delay and the traffic emissions via regression analysis. The delay is changed from 0 to 40 seconds in steps of 0.1. When the speed decreases from *v*_*f*_ = 70*km*/*h* to zero, the required time is calculated using Eq 18:

$$t=v_{f}/a_{\alpha }\left(t\right)$$ (18)

Therefore, when the control delay is between 0 and 2 s, the vehicle will decelerate and then accelerate; it will not be idle. For example, when the control delay is 1.5 s, then the vehicle decelerates with *a*_*d*_(*t*) = 2.74*m*/*s* ^2^, and the speed at time 1.5 s can be calculated using:

$$v=v_{f}-a_{d}(t)\times t$$ (19)

The VSP value when a vehicle is decelerating or accelerating to *v*_*f*_ = 70*km*/*h* can also be calculated as follows; this process requires an amount of time t:

$$\tau =\text{ln}\left(v_{a}\left(t\right)/\left(\frac{b}{a}\right)+1\right)/a$$ (20)

$$S_{a}\left(t\right)=\frac{b}{a^{2}}\left(e^{at}-at-1\right)$$ (21)

Based on the vehicle trajectory, the emissions calculated based on VSP is 7.106785597 mg.

Using the above Eqs 16–21, the threshold of vehicle delay is obtained as follows:

$$D_{c}=(t_{d}-\frac{S_{d}}{v_{f}})+(t_{a}-\frac{S_{a}}{v_{f}})=2.0s$$ (22)

**Fig 2:**
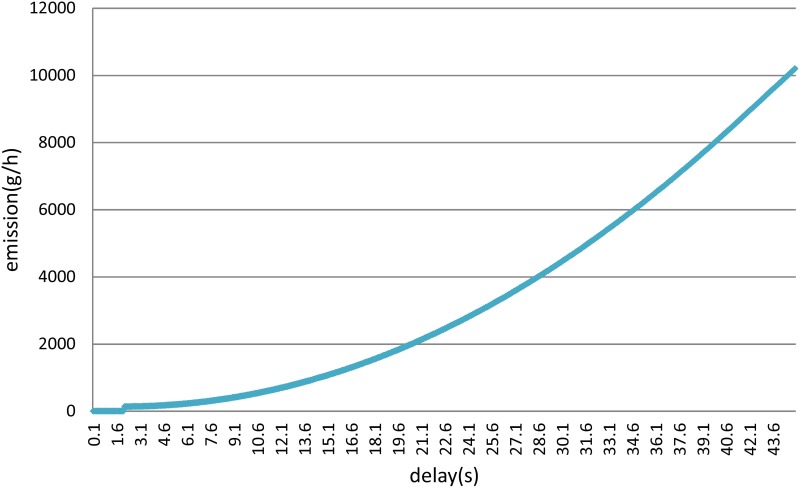
Relationship between delay and emissions.

When the control delay is less than 2.0 seconds, the vehicle is not idle. When the control delay is more than 2 seconds, in addition to completing the movement processes of acceleration and deceleration, an idle process is also performed. As shown in Fig 2, emissions tend to increase with the control delay.

As a result, the relationship between the delay and the associated emissions is described by Eq 23:

$$y=0.0538x^{2}-1.6877x+106.07$$ (23)

where *y* is the amount of emissions produced, and *x* is the value of the control delay.

The dissipation time during queuing while the green signal is illuminated is calculated as follows:

$$t_{B1}=ER\cdot q_{d}/(s-q_{d})$$ (24)

where *ER* represents the effective green signal time, *q*_*d*_ represents the arrival flow rate and *S* represents the saturation flow rate.

The total time that a vehicle is blocked from movement at the intersection is the sum of the red signal time and the time that queuing dissipates during the green signal:

$$t_{B2}=ER+t_{B1}=ER\cdot S/(s-q_{d})$$ (25)

As a result, the control delay of the vehicles is calculated as:

$$q_{B}=q_{d}\cdot \frac{t_{B2}}{CL}$$ (26)

where *CL* is the cycle time.

Thus, the emissions of one direction’s traffic flow at the intersection under the control of the traffic signal control is obtained as follows:

$$Em_{i}\left(ER_{i},CL\right)=\int_{0}^{ER_{i}}\left(\frac{1}{ER_{i}}\cdot y\cdot q_{B}\right)dx=\frac{1}{ER_{i}}\times (0.538\times \frac{1}{3}\times ER_{i}{}^{3}-1.6877\times \frac{1}{2}ER_{i}{}^{2}+106.07\times ER_{i})\times q_{B}$$ (27)

where *Em*_*i*_(*ER*_*i*_,*CL*) represents the traffic emissions of the traffic flow *i* within one hour.

### Delay calculation

This study uses the HCM2000 delay equation to calculate the intersection vehicle delay[38, 55]:

$$d=d_{1}\text{+}d_{2}$$ (28)

$$d_{1}=\frac{0.5C{\left(1-\lambda \right)}^{2}}{1\text{-}[\text{min}(1,X)\lambda ]}$$ (29)

$$d_{2}=900T\left[\left(X-1\right)+\sqrt{{\left(X-1\right)}^{2}+\frac{8kIX}{cT}}\right]$$ (30)

where *d* is the control delay (s); *d*_1_ is the even delay (s); *d*_2_ is the incremental delay (s); *λ* is the split; *X* is the saturation; *C* is the cycle time; *c* is the capacity (vehicles/h); *T* is the duration of the analysis (h); and *k* is the incremental delay correction factor of sensor control, where *k* is equal to 0.5 for a given signal cycle; and *I* is the incremental delay correction factor for vehicles to change lanes in the upstream channel, and the value of this factor is 1.0.

Using the above information, the total delay of the intersection can be obtained:

$$D=\sum_{j=1}^{n}\left(d_{j}\times q_{j}\right)$$ (31)

where *d*_*j*_ is the average vehicle control delay of *j*_th_ traffic signal phase of the intersection; *q*_*j*_ is the traffic flow of *j*_th_ traffic signal phase of the intersection.

### Overall operating efficiency model for intersections

The key to a multi-objective optimization problem is determining how to combine and achieve various objectives. To achieve optimal control, we transform the multi-objective programming problem described above into a traditional single-objective programming problem using the utility optimization model. Different units of delay and emission are also considered, and the following model is established after normalization:

$$PI=\alpha \cdot \frac{D}{D_{0}}+\beta \cdot \frac{E}{E_{0}}$$ (32)

where *PI* is the comprehensive benefit function value; *α* is the weight of the delay; *β* is the weight of emissions; *D* and *E* are the total delay time and total amount of emissions of the intersection, respectively; and *D*_0_ and *E*_0_ are the initial values of the delay of the signal timing and the emissions, respectively.

The value of *PI* varies based on *α* and *β*. When *α* is equal to 1 and *β* is equal to 0, the operational benefit of a given intersection depends only on the length of the delay.

### Solution algorithm

A proper cycle time can ensure that vehicles move through an intersection in an orderly and smooth manner. When traffic flow is slow, the cycle length should be small (i.e., generally no less than 15n, where n is the phase number of the intersections) because a short period of time can cause vehicles to queue in the intersection, thus affecting traffic safety. When traffic flow is high, the capacity should be the highest priority, and the cycle time should be long but not more than 200 seconds, considering the psychology of driver boredom and the tendency of vehicle drivers to run red lights. As a result, the value of the cycle time should be defined as follows:

15n≤C≤200s (33)

When the saturation satisfies 0.8≤*V*/*C*≤1, the operating conditions can be regarded as good, and the optimization function *PI*(*C*) can be constrained as follows:

$$x_{i}=\frac{y_{i}}{\lambda_{i}}$$ (34)

$$0.8\leq x_{i}\leq 1.0$$ (35)

**Fig 3:**
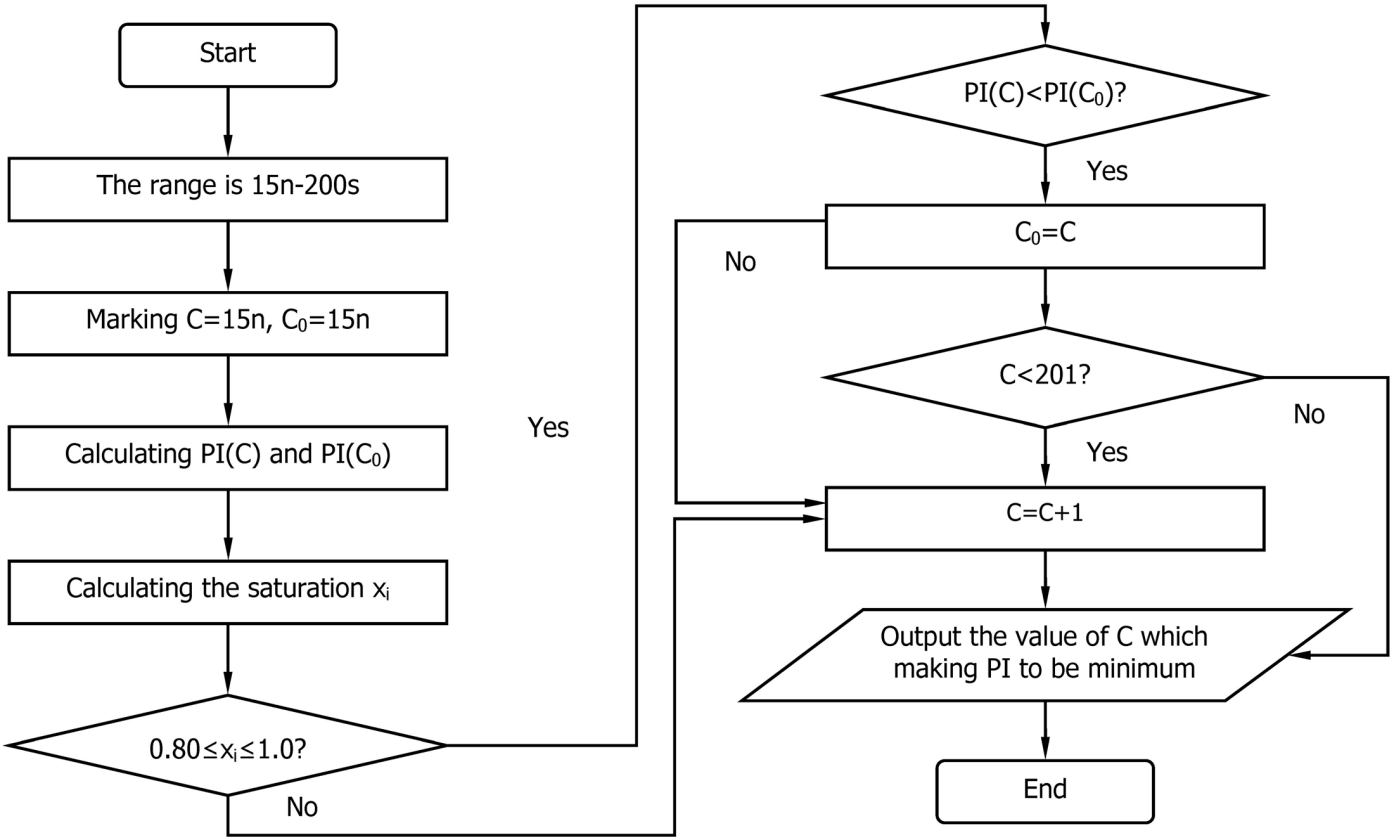
Cycle optimization flow chart.

In this study, a comprehensive enumeration method is used to optimize the model. The specific optimization process used is shown in Fig 3.

Step 1. Define the search range of cycle *C* as 15n-200*s*, where n is the number of phases.
Step 2. Define the search interval of cycle *C* as 1*s*.
Step 3. Set *C* = 15*n* and *C*_0_ = 15*n*.
Step 4. Calculate the value of *PI*(*C*_0_).
Step 5. Calculate the value of *PI*(*C*).
Step 6. Calculate the saturation *x*_*i*_. If 0.8≤*x*_*i*_≤1.0, then go to Step 7; otherwise, go to Step 9.
Step 7. If *PI*(*C*)<*PI*(*C*_0_), then *C*_0_ = *C*.
Step 8. If *C*<201, then go to Step 9; otherwise, end the process.
Step 9. Perform *C* = *C*+1.
Step 10. Go Step 4.

After optimization, the cycle that meets the minimum value of *PI* can be obtained. Next, use the principle of equal saturation to determine the signal timing design for the intersection and choose the optimized timing instead of the original timing. As mentioned above, a smaller value of *PI* corresponds to better control of the emissions and delay; thus, we can effectively optimize the signal timing of a given intersection.

## Results

### Input data

**Fig 4:**
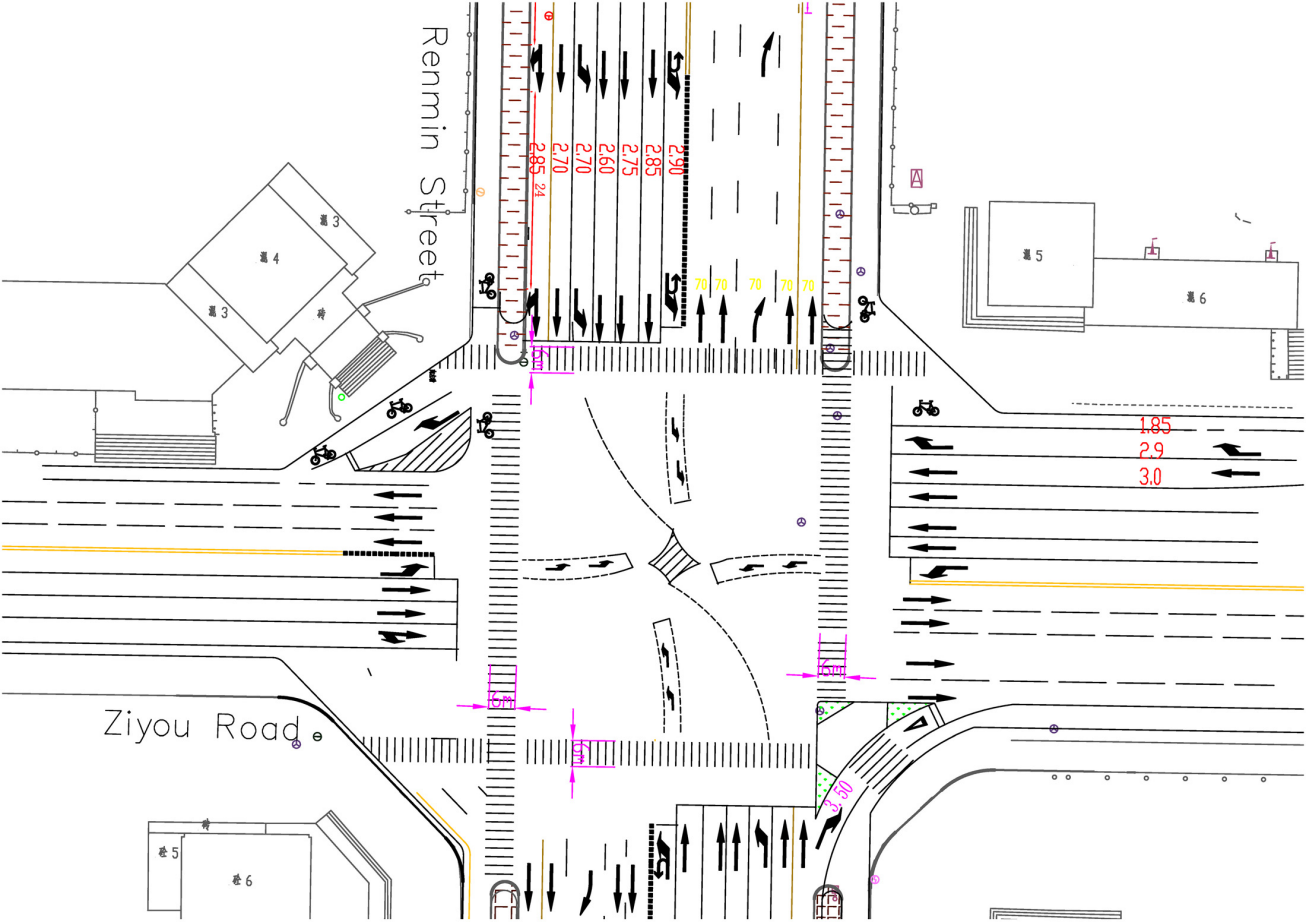
Geometric characteristics of the Renmin-Ziyou intersection in Changchun city.

To verify the method described above, this study considers the Renmin-Ziyou intersection in Changchun city. The geometric, signal control and traffic flow information of this intersection were collected from the field. The geometric features of the intersection are shown in Fig 4. Traffic flow data were obtained from loop-coil vehicle detectors and video camera vehicle detectors from August 10 to 23, 2014. The traffic flow data were provided and permitted to use by Changchun traffic police detachment of public security. The interval between data collection events was 5 minutes. These detectors collect various traffic parameters, including traffic volume, speed and occupancy rate. The traffic flow data and intersection features were input into the microscopic traffic simulation software (VISSIM) to evaluate the traffic signal control performances and the average vehicle emissions.

During the investigation process, the traffic was determined to be primarily composed of buses and cars on the road; these types of vehicles are represented by a cart and a car, respectively. The conversion coefficient of the cart is 2.0, and the conversion coefficient of the small car is 1.0[34, 39]. In this study, the average speed of the vehicles is assumed to be 40 km/h, and the length of the approach is assumed to be 100 m. Based on the above information, the traffic volume of each phase’s key traffic flow is obtained. The straight flow from north to south is 327/h, the left-turn flow from north to south is 244/h, the straight flow from west to east is 374/h, and the left-turn flow from west to east is 411/h. After obtaining the basic information of the intersection, we applied the model proposed in this study to obtain the initial delay and emissions of the intersection: the initial total delay was determined to be 8.2859×10^5^ seconds, and the initial emissions were determined to be 2.3969×10^4^ grams per hour.

### Simulation parameters

**Fig 5:**
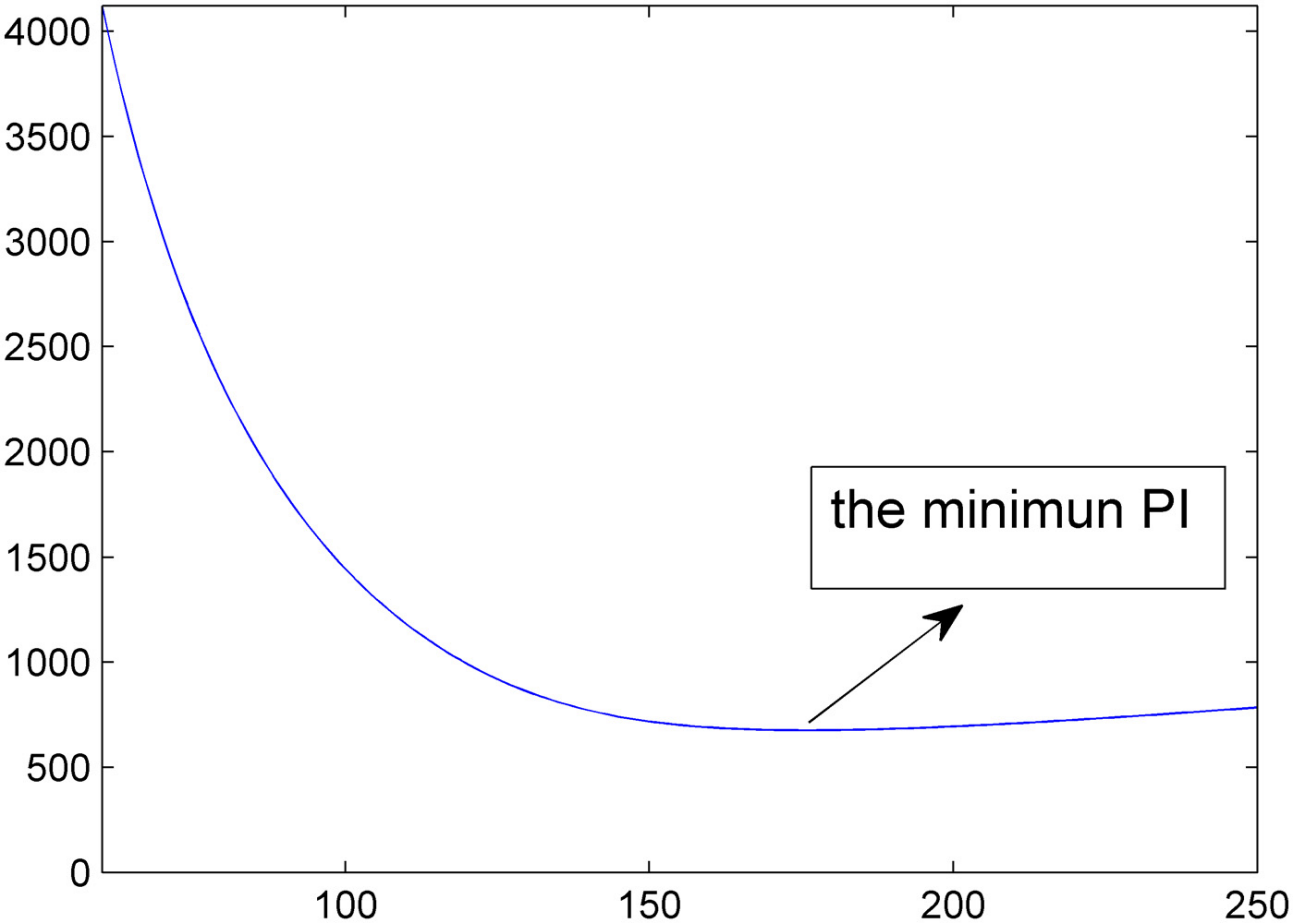
*PI* as the cycle changes.

As mentioned above, the enumeration method is used to optimize the model and to limit it within the saturation and cycle. To improve the optimization efficiency, this study used MATLAB to solve the model. In the programming process of the enumeration methods, the calculation procedures for *PI* are used many times. As a result, this study modularizes this portion of the programming and uses the function *PI* = *zibianhanshu*(*C*). Fig 5 shows the changes in *PI* as the cycle changes, and a cycle that achieves the minimum value of *PI* is identified, as expected. Next, this study determined this cycle by applying the method of enumeration.

**Fig 6:**
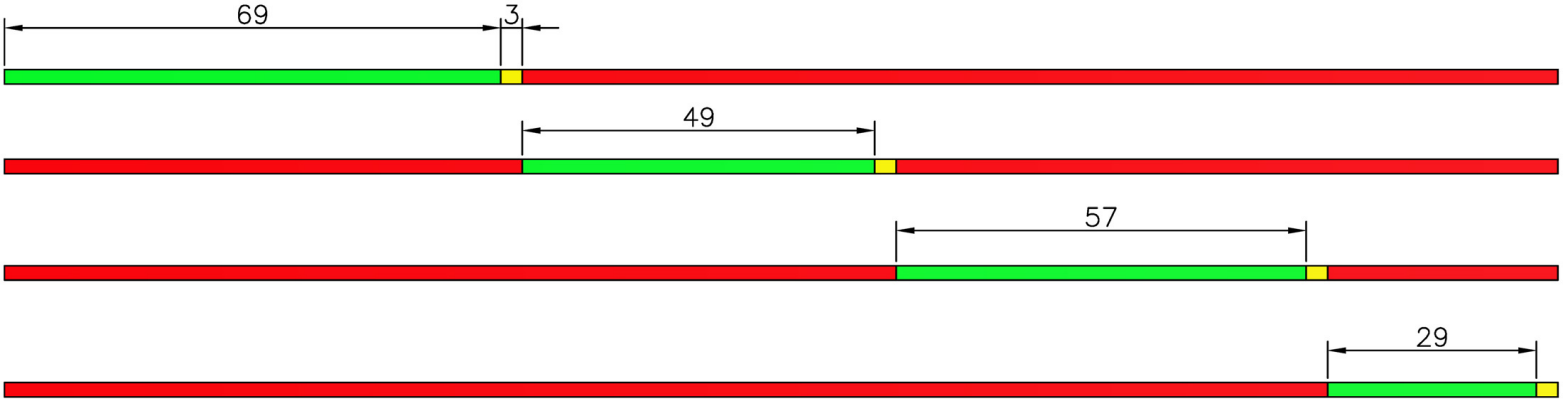
Original timing plan.

**Fig 7:**
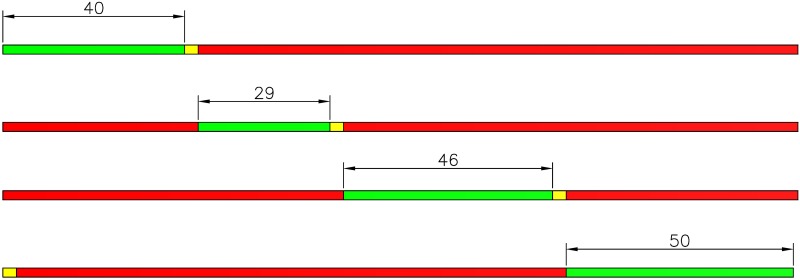
Optimized timing plan.

Based on the optimization algorithm and real survey data, *C* = 176 yields the minimum *PI*. This optimization cycle is less than the original (216 s) and within the limits defined above, which allows the driver to accept the longer red light without too much impatience. Next, the equal saturation principle is used to calculate the optimal timing plan. The original timing plan and the optimized timing plan are shown in the Figs 6 and 7 as below.

### Simulation implementation

**Fig 8:**
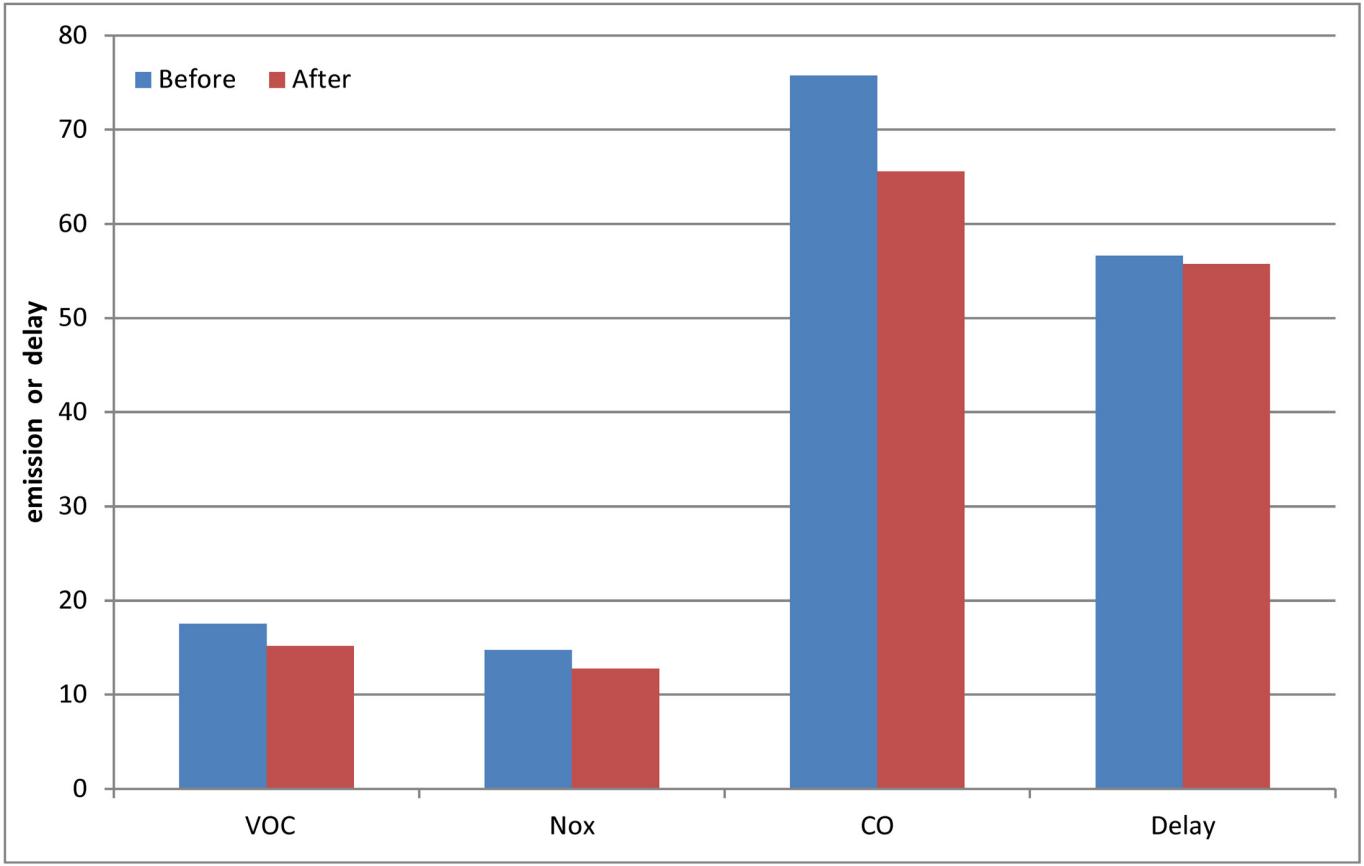
Contrast of related indices before and after optimization.

Using VISSIM to simulate the intersection before and after changing the traffic signals, the effect of optimizing the delay and emissions is determined. The delay and emissions are compared based on the simulations before and after changing the signal control; these results are shown in Tables 2 and 3 and Fig 8.

**Table 2:** Contrast of the delay before and after optimization.

	Original plan	Optimized plan
delay (s)	56.62333333	55.76789474

**Table 3:** Contrast of the emissions before and after optimization.

	VOC	NO_x_	CO
Before	17.54935417	14.7330625	75.72191667
After	15.19551394	12.75681531	65.56557233

From the data in the table, it is shown via comparison that both the delay and emissions are improved after optimization. The results show that changing the signal timing is effective; thus, using the model proposed in this study to optimize timing can improve the running status of an intersection and achieve the goal of this paper. The model considers the emissions and delay factors concurrently, improving the efficiency of the traffic and the environment.

## Conclusions

Effective traffic signal control is required to ensure smooth and orderly operation of urban road traffic; such control is currently a popular research topic of many scholars. However, previous studies primarily focused on reducing traffic congestion and ignoring the influence of traffic on the environment. With the growing consciousness of people regarding environmental protection, a traffic signal control scheme that considers environmental effects is required; therefore, this article focused on the development of such a scheme. A multi-objective study of traffic signal control was performed in view of the present development trend of traffic signal control; emissions were regarded as one of the goals for improving the operation of intersections to optimize the intersections based on vehicular delay.

Urban traffic signal control is a complex topic. The development described in this study addresses only one aspect of the topic (i.e., the primary stage) with results that are only applicable for a single intersection when considering both emissions and delay. Related research for arterial coordination and regional coordination was not considered in this article; such topics will be considered in a follow-up study and discussed in depth.
